# The TRPV3 channel of the bovine rumen: localization and functional characterization of a protein relevant for ruminal ammonia transport

**DOI:** 10.1007/s00424-020-02393-2

**Published:** 2020-05-26

**Authors:** Franziska Liebe, Hendrik Liebe, Sabine Kaessmeyer, Gerhard Sponder, Friederike Stumpff

**Affiliations:** 1 Institute of Veterinary Physiology, Freie Universität Berlin, Oertzenweg 19b, 14163 Berlin, Germany; 2 Department of Biology, Chemistry, and Pharmacy, Freie Universität Berlin, Arnimallee 22, 14195 Berlin, Germany; 3 Institute of Veterinary Anatomy, Freie Universität Berlin, Koserstraße 20, 14195 Berlin, Germany

**Keywords:** TRPV3, Ammonia transport, Microelectrode, Climate gas, Rumen, *Xenopus* oocyte

## Abstract

Large quantities of ammonia (NH_3_ or NH_4_^+^) are absorbed from the gut, associated with encephalitis in hepatic disease, poor protein efficiency in livestock, and emissions of nitrogenous climate gasses. Identifying the transport mechanisms appears urgent. Recent functional and mRNA data suggest that absorption of ammonia from the forestomach of cattle may involve TRPV3 channels. The purpose of the present study was to sequence the bovine homologue of *TRPV3* (*bTRPV3*), localize the protein in ruminal tissue, and confirm transport of NH_4_^+^. After sequencing, bTRPV3 was overexpressed in HEK-293 cells and *Xenopus* oocytes. An antibody was selected via epitope screening and used to detect the protein in immunoblots of overexpressing cells and bovine rumen, revealing a signal of the predicted ~ 90 kDa. In rumen only, an additional ~ 60 kDa band appeared, which may represent a previously described bTRPV3 splice variant of equal length. Immunohistochemistry revealed staining from the ruminal *stratum basale* to *stratum granulosum*. Measurements with pH-sensitive microelectrodes showed that NH_4_^+^ acidifies *Xenopus* oocytes, with overexpression of bTRPV3 enhancing permeability to NH_4_^+^. Single-channel measurements revealed that *Xenopus* oocytes endogenously expressed small cation channels in addition to fourfold-larger channels only observed after expression of bTRPV3. Both endogenous and bTRPV3 channels conducted NH_4_^+^, Na^+^, and K^+^. We conclude that bTRPV3 is expressed by the ruminal epithelium on the protein level. In conjunction with data from previous studies, a role in the transport of Na^+^, Ca^2+^, and NH_4_^+^ emerges. Consequences for calcium homeostasis, ruminal pH, and nitrogen efficiency in cattle are discussed.

**Electronic supplementary material:**

The online version of this article (10.1007/s00424-020-02393-2) contains supplementary material, which is available to authorized users.

## Introduction

Ammonia in its two forms (NH_3_ and NH_4_^+^) plays a central role in the interconversion of amino acids for protein metabolism, requiring rapid transport across membranes of cells and organelles. Surprisingly, little information is currently available concerning the transport of this metabolite by epithelia of the gut. Given that more than half of the ammonia found in peripheral blood is of gastrointestinal origin [31], understanding the mechanisms responsible for ammonia absorption might help with a better management of hyperammonemia in patients suffering from hepatic disease. However, the most urgent task may be to find strategies to reduce the vast amounts of nitrogen that are excreted by livestock worldwide, leading to human respiratory problems, eutrophication, and climate change [28]. Livestock production represents the largest anthropogenic source of the highly potent climate gas N_2_O [56]. The nitrogen in this compound originates from dietary protein that is broken down to ammonia in the gut. This ammonia can be utilized for microbial protein synthesis, but unfortunately, the larger fraction of this toxin is absorbed, converted to urea, and excreted into the environment with disastrous consequences.

So why are the losses of ammonia from the gut so high? As recently as two decades ago, it was widely believed that epithelial ammonia transport occurred by simple diffusion of the uncharged form (NH_3_) through the lipid bilayer of the cell membrane [57]. However, like water, NH_3_ has a strong dipole moment and it has become increasingly clear that proteins are required to mediate transport. In the collecting duct of the kidney, it has been established that aquaporins are required for the transport of water. Likewise, Rh-glycoproteins are necessary to mediate ammonia transport. The apical ammonia transporter RhCG is considered to be highly selective for NH_3_ while the substrate (NH_3_ or NH_4_^+^) of the basolateral RhBG has not yet been clarified [12, 24, 35]. Far less information is available on intestinal absorption of ammonia. In analogy to the collecting duct, electroneutral apical uptake of NH_3_ via RhCG and basolateral efflux via RhBG has been proposed for the intestine of mice [25] or toadfish [10]. Conversely, exchange of NH_4_^+^ with H^+^ via sodium-proton exchange (NHE) has been suggested in rat colon [13]. In pig caecum and trout intestine, NH_4_^+^ is taken up in an unclear, electrogenic mechanism [46, 52].

Comparatively, more information is available concerning ammonia transport across the forestomach of ruminants. The interest is old [22, 33] and triggered by the low protein efficiency of cattle [20, 26]. In the largest of the forestomachs, the rumen, the cellulose-rich diet is broken up into digestible components by resident microbial populations. Microbial protein is produced from any nitrogen source available, including ammonia and urea [1, 43]. Unfortunately, large quantities of ammonia are absorbed from the rumen before they can be utilized. However, the ruminal epithelium expresses transport proteins through which urea can reenter the rumen and serve as a source of nitrogen for microbial protein synthesis [2, 43, 50, 65]. Since this protein can be fully digested in the following parts of the gastrointestinal tract, this recycling of nitrogen allows ruminants to subsist on low-grade, poorly digestible fodder while yielding milk and meat. Problems emerge when cattle are fed large quantities of high-quality protein required for maximal yields in industrial farming. In this scenario, blood urea levels rise and nitrogen recycling leads to secretion of some 10 mol day^−1^ of urea into the rumen, where it is degraded to ammonia, reabsorbed, and again converted to urea and resecreted, requiring ~ 40 mol day^−1^ of ATP for hepatic detoxification. Despite recycling, up to 70% of dietary nitrogen is eventually excreted into the environment with urine and feces [20].

Clearly, a clarification of the mechanism responsible for the high efflux of ammonia from the rumen is overdue. The expression profile in the ovine rumen does not appear to support the involvement of either RhCG or RhCB [65]. Systematic studies of ruminal transport of ammonia have established that electroneutral transport of NH_3_ is predominant at an alkaline pH of 7.4. At a physiological pH of 6.4, transport primarily involves electrogenic uptake of NH_4_^+^ [3, 4, 7, 22, 40]. Thus, studies with microelectrodes and in Ussing chambers have shown that exposure to NH_4_^+^ depolarizes both the apical membrane and the intact ruminal epithelium in toto with acidification of the cytosolic space [32, 45], resulting in stimulation of NHE [3, 4].

Based on both functional evidence and mRNA data, the bovine homologue of the non-selective transient receptor potential cation channel, subfamily V, member 3 (bTRPV3) has recently emerged as a candidate for uptake of cations from the rumen, including NH_4_^+^ and Ca^2+^ [40, 45]. Like most other members of the large family of TRP channels [39], both the human and the bovine homologue of TRPV3 are known to be permeable to a number of monovalent and divalent cations, including Na^+^ and Ca^2+^ [48, 63]. Given that certain plant-derived compounds modulate the activity of TRPV3 [55], this has implications both for the development of new drugs and for new feeding strategies [8]. Ruminants absorb considerable amounts of Ca^2+^ from the rumen to meet the high demand involved in milk production [61]. Based on electrophysiological data, a ruminal channel for Ca^2+^ has long been postulated [27, 62], although the typical epithelial calcium channels TRPV5 and TRPV6 are not expressed in the ruminal epithelium [45, 60]. Intriguingly, TRPV3 channels are activated by intracellular protons [36], which may explain the well-documented stimulatory effects of short-chain fatty acids on ruminal calcium uptake [61].

In contrast to a wealth of data concerning the permeability of TRPV3 to Ca^2+^ [36, 39, 55, 63], the permeability of TRP channels in general and TRPV3 in particular to NH_4_^+^ had never been investigated before our recent patch-clamp study of HEK-293 cells [48]. One goal of the present study was to confirm transport of NH_4_^+^ by bTRPV3 using a different expression system (*Xenopus* oocytes) and a different method (pH-sensitive microelectrodes), both as established in studies of other ammonia transporters [12, 24]. Furthermore, a confirmed sequence and detection on the protein level are lacking. The major aim of the current study was therefore to properly sequence the bTRPV3, to establish a suitable antibody, and finally to localize the channel in the ruminal epithelium.

## Materials and methods

### Animal welfare

The maintenance and surgical treatment of *Xenopus laevis* frogs was in accordance with the guidelines of German legislation, with approval by the animal welfare officer for the Freie Universität Berlin and under the governance of the Berlin Veterinary Health Inspectorate (Landesamt für Gesundheit und Soziales Berlin, permit G0025/16).

Bovine ruminal epithelium was obtained from Holstein-Friesian cattle slaughtered for meat production in a commercial abattoir (Beelitz, Germany), also under control of the German authorities.

### Ruminal tissue

Pieces of the bovine rumen were removed about 10 min after death and immediately stripped, rinsed twice with PBS, and dissected into pieces of 1 to 2 cm^2^. Samples were shock-frozen in liquid nitrogen and stored at − 80 °C or transferred into formaldehyde solution (Roti®-Histofix 4%, Carl Roth, Karlsruhe, Germany). Unless indicated otherwise, only ventral rumen was used as the locus with maximal absorptive capacity.

### Sequencing and cloning of *bTRPV3*

Shock-frozen bovine ruminal epithelium was used for mRNA extraction with subsequent reverse transcription to cDNA, which was used to sequence the bovine representative of the *TRPV3* channel (*bTRPV3*). The construct was tagged with a streptavidin (Strep) tag (ShineGene Bio-Technologies Inc., Shanghai, China), which was placed at the N terminus to prevent possible interference with a C-terminal PDZ binding motif found in some TRP channels [41]. This Strep-*bTRPV3* construct was then subcloned into pIRES2-*AcGFP*1 (Takara BioEurope, Saint-Germain-en-Laye, France) or into pcDNA5/TO (Life Technologies, Darmstadt, Germany) as described previously [48]. Cells successfully transfected with Strep-*bTRPV3*-pIRES2-*AcGFP*1 showed green fluorescence.

For expression of *bTRPV3* in *Xenopus* oocytes, the restriction enzymes PasI and XbaI were used to replace the last 716 bp of the Strep-*bTRPV3*-pcDNA5/TO construct with a 713-bp fragment lacking the stop codon. The resulting construct was then cut out from Strep-*bTRPV3*-pcDNA5/TO and subcloned into pGEM-HE-MCS (kindly donated by Prof. Blanche Schwappach, Georg-August-Universität, Göttingen, Germany) via the restriction sites HindIII and XbaI. The restriction enzyme MluI was used for linearization and RiboMAX Large Scale RNA Production System-T7 (Promega, Mannheim, Germany) was used for in vitro transcription to cRNA according to the manufacturer’s instructions.

### Harvesting and injection of *Xenopus* oocytes

*Xenopus laevis* oocytes were obtained and prepared as described by Vitzthum et al. [54] After surgical removal, ovarian lobes were placed in oocyte Ringer’s solution (180 mOsm kg^−1^ adjusted with D-mannitol) [54], shaken mechanically for 90 min, and transferred into calcium-free oocyte Ringer’s solution for 10 min. Defolliculated stage V–VI oocytes were stored in oocyte culture solution at 16 °C until the following day, when they were injected with 50 nL RNAse free water containing 15–30 ng of *bTRPV3*-Strep cRNA (WPI Nanoliter 2010, World Precision Instruments, Sarasota, FL, USA). Control oocytes were injected with 50 nL RNAse free water. Injected oocytes were incubated for at least 3 days in modified low-sodium oocyte culture solution before use in experiments (in mmol L^−1^: 80 N-methyl-D-glucamine chloride (NMDGCl), 5 NaCl, 5 4-(2-hydroxyethyl)-1-piperazineethanesulfonic acid (HEPES), 2.5 2-Oxopropanoic acid, 1 KCl, 1 CaCl_2_, 1 MgCl_2_, 50 units mL^−1^ penicillin, 0.05 mg mL^−1^ streptomycin, pH 7.4 adjusted with tris (hydroxymethyl) aminomethane (Tris), 223 mOsm kg^−1^ adjusted with D-mannitol).

### Cell culture and transfection of HEK-293 cells

HEK-293 cells (DSMZ, Braunschweig, Germany, 2016/06/08) were cultivated at 37 °C in Dulbecco’s modified Eagle’s medium (FG 0445) supplemented with 10% fetal bovine serum and 100 units mL^−1^ of penicillin and streptomycin (all Biochrom, Berlin, Germany). Polyethylenimine (PEI, linear, MW 25000, Polysciences, Inc., Hirschberg an der Bergstrasse, Germany) was used to transiently transfect the cells with the Strep-*bTRPV3*-pIRES2-*AcGFP*1 vector or with the empty pIRES2-*AcGFP*1 vector as control (http://www.cytographica.com/lab/PEItransfect.html). Experiments were performed 48 h after transfection.

### Immunoblotting

Both the solvents and the samples were cooled throughout the experiments to minimize protein degradation.

#### Bovine rumen

RIPA buffer (500 μL; in mmol L^−1^: 25 HEPES, 2 EDTA, 25 NaF, protease inhibitor (cOmplete™, mini, Roche, Basel, Switzerland), 1% sodium dodecyl sulfate (SDS)) was added to the defrosted tissue (200 mg) together with two metal beads. The tissue was homogenized in a mixer mill (30 × 2 min; MM 200, Retsch GmbH, Haan, Germany), followed by a clarifying spin (15 min, 20,000 g, 4 °C). The supernatant containing the protein was transferred into a new tube.

#### *Xenopus* oocytes

After the removal of the culture medium, ten oocytes of each transfected group were lysed mechanically in oocyte lysis buffer (500 μL; in mmol L^−1^: 5 MgCl_2_, 5 NaH_2_PO_4_, 1 EDTA, 80 sucrose, pH 7.4 (Tris)). The suspension was centrifuged (200 rpm, 10 min, 4 °C) and the supernatant was transferred to a new tube, after which the centrifugation step was repeated. The supernatant was then centrifuged a third time (13,000 rpm, 40 min, 4 °C). The precipitate was suspended in fresh oocyte lysis buffer (40 μL).

#### HEK-293 cells

After washing with phosphate-buffered saline (PBS), HEK-293 cells were harvested mechanically by scraping in PBS. After centrifugation (500 g, 5 min), the cell pellet was suspended in PBS (1 mL) and transferred into a new tube. PBS was removed via centrifugation (700 g, 4 min) and the cell pellet was suspended in RIPA buffer (100 μL). Lysis was performed for 30 min with gentle agitation and 5 min in an ultrasound bath, followed by a clarifying spin (20 min, 15,000 g), and supernatant with protein was stored at − 80 °C.

The protein concentration of each suspension was determined using the Pierce™ 660 nm protein assay kit (Thermo Fischer Scientific, Waltham, MA, USA). Proteins were denatured in SDS sample loading buffer (10%) and electrophoresed on polyacrylamide gels (7.5%, SDS-PAGE) in Tris-Glycine buffer (0.1% SDS). Electroblotting was performed onto polyvinylidene difluoride membranes (PVDF, Immun-Blot®, Bio-Rad Laboratories GmbH, Munich, Germany) in Tris-Glycine buffer (0.3% SDS, 20% methanol, 4 °C).

#### Antibodies against bTRPV3

After the first attempts to stain ruminal tissues with a commercial antibody against the human homologue (ab63148, abcam, Cambridge, UK) had failed, epitopes of several commercial antibodies against the human TRPV3 channel were aligned with the sequence of the bovine homologue. A primary mouse antibody directed against an epitope (AA 458-474) from the first extracellular loop of the human TRPV3 channel was selected as the most promising candidate and used at a dilution of 1:3000 (ID: ABIN863127, antibodies-online GmbH, Aachen, Germany). The human epitope (SYYRPREEEA**I**PHPLA) showed almost complete homology with the bovine sequence (SYYRPREEEA**L**PHPLA). For control purposes, a primary mouse antibody directed against the Strep-tag of the clones (1:2500; ID: 34850, Qiagen, Hilden, Germany) was used. Horseradish peroxidase conjugated secondary antibody (anti-mouse, 1:1000; Cell Signaling Technology, Frankfurt, Germany) was used to detect the primary antibodies on the membrane. Proteins were visualized by use of the Clarity Western ECL Substrate (Bio-Rad Laboratories GmbH, Munich, Germany).

These experiments yielded an additional prominent band below the expected molecular weight of full-length bTRPV3. To test for binding via the domains specific for the target epitope [9], additional immunoblots with primary TRPV3 antibody were performed in the presence and absence of the peptide used to produce the TRPV3 antibody (referred to as specific immunizing peptide or SIP, smc 334d_peptide, antibodies online GmbH, Aachen, Germany) and a control peptide (CP, anti SLC41A3, Santa Cruz Biotechnology, Heidelberg, Germany), all at 1:1000. Exposure time was optimized via the ImageLab software (BioRad), which was also used to calculate the relative quantities (see Supplement).

### Immunohistological staining

All preparation steps were performed as described in detail in the Supplement or as described in [51]. Samples from both the ventral and the dorsal rumen were stained with primary antibody diluted in goat serum (5% in PBS; PAN-Biotech GmbH, Aidenbach, Germany) according to Table 1 (4 °C, overnight). Secondary antibody controls were performed with goat serum (5% in PBS) only. To test for specificity of binding, adjacent slices from the same sample of ruminal tissue were incubated in parallel either with the primary mouse TRPV3 antibody only or with a mix of this antibody and its corresponding antigenic peptide (SIP) (Table 1). Images were obtained using a confocal laser-scanning microscope (LSM 510, Axiovert200M, Zeiss, Jena, Germany) at 405, 488, and 543 nm.

**Table 1:** Primary and secondary antibodies used for immunohistochemical staining

	HEK-293 cells	Ruminal tissue	*Xenopus* oocytes
Primary antibody 1	mouse TRPV3 antibody (1:1000)^§^	mouse TRPV3 antibody (1:1000)^§^	mouse TRPV3 antibody (1:1000) ^§^
Primary antibody 2		rabbit claudin-4 antibody (1:250)^&^	
Secondary antibody 1	594 goat anti-mouse IgG (1:1000 )*	488 goat anti-mouse IgG (1:1000 )*	488 goat anti-mouse IgG (1:1000 )*
Secondary antibody 2		594 goat anti-rabbit IgG (1:1000 )*	
Immunizing Peptide		smc-334d_peptide (1:330)^#^	

^§^ABIN863127, antibodies-online GmbH, Aachen, Germany

*Alexa Fluor®, Thermo Fischer Scientific, Waltham, MA, USA

^&^AB53156, Abcam, Cambridge, UK

^#^smc-334d_peptide, antibodies-online GmbH, Aachen, Germany

### Double-barrelled pH-sensitive microelectrode measurements

pH-sensitive microelectrodes were prepared as described in detail previously [2] and in the Supplement. The potential difference between the pH-sensitive barrel and the reference barrel was used to determine the intracellular pH (pH_i_), while the potential difference between the reference barrel and the ground signal from the bath corresponded to the membrane potential (*U*_mem_). Electrodes were calibrated before and after each measurement (23 °C). After each measurement of an oocyte expressing bTRPV3, a control oocyte was measured.

All microelectrode solutions were adjusted to an osmolality of 223 mOsm kg^−1^ using D-mannitol, and were adjusted to pH 7.4 (Tris) and contained (in mmol L^−1^) 5 HEPES, 1 CaCl_2_, 1 MgCl_2_, and 1 KCl. The following solutions were used (in mmol L^−1^): NaCl (85 NaCl), KCl (81 KCl, 5 NaCl), NaGlu (80 sodium D-gluconate (NaGlu), 5 NaCl, 10 NMDGCl), NH_4_Cl (5 NaCl, 80 NH_4_Cl), NH_4_Cl-EDTA (5 NaCl, 80 NH_4_Cl, 5 NMDGCl, no CaCl_2_ and no MgCl_2_), NMDGCl (80 NMDGCl, 5 NaCl), and NaCl-6.4 (85 NaCl, 5 2-(*N*-morpholino) ethanesulfonic acid (MES), pH 6.4, no HEPES).

### Inside-out patch-clamp experiments

Single-channel experiments were performed as previously described [23, 48] in a continuously perfused bath chamber at 23 °C. Pipettes were pulled with a DMZ Universal Puller (Zeitz Instruments, Munich, Germany). Currents were recorded by an EPC 9 patch-clamp amplifier (HEKA Electronic, Lambrecht, Germany) using the Patchmaster Software (HEKA Electronic). Data were sampled at 10 kHz and filtered at 250 Hz. Currents were clamped at the potentials − 60 to + 60 mV in 10 mV steps for 6 s each. After each oocyte overexpressing bTRPV3, a control oocyte was measured.

All single-channel patch-clamp solutions were adjusted to an osmolality of 223 mOsm kg^−1^ using D-mannitol and had a pH of 7.4 adjusted with Tris and HCl. Initially, oocytes were incubated (5–10 min) in oocyte Ringer (in mmol L^−1^: 96 NaCl, 5 HEPES, 2.5 2-Oxopropanoic acid, 1 KCl, 1 CaCl_2_, 1 MgCl_2_)_._ Oocytes were then placed in a cell culture dish under a dissecting microscope and D-mannitol was added incrementally until the vitelline membrane began to dissociate so that it could be manually removed with sharpened forceps. Care was taken not to damage the plasma membrane. After allowing the stripped oocyte to settle on the glass bottom of a conventional flow chamber over an inverted microscope (Axiovert.A1, Zeiss), a seal was formed and a membrane patch excised and measured in the usual manner. Solutions were based on those used by Doerner et al. [18] and contained (in mmol L^−1^) 20 HEPES, 5 CsCl, 1 ethylene glycol-bis(β-aminoethyl ether)-*N*,*N*,*N*′,*N*′-tetraacetic acid (EGTA), and 1 KCl. The pipette and the NH_4_Cl bath solution additionally contained NH_4_Cl (96 mmol∙L^−1^). In NaCl, KCl, and NMDGCl bath solutions, NH_4_^+^ was replaced by the same amount of Na^+^, K^+^, or NMDG^+^ respectively. NH_4_Glu bath solution substituted 81 mmol L^−1^ NH_4_Cl with NH_4_-gluconate.

### Data analysis

Data processing was performed using Igor Pro 6.2.2.2 (WaveMetrics Inc., Lake Oswego, USA) and Sigma Plot 11.0 (Systat Software 11.0, Erkrath, Germany). All potentials were corrected for liquid junction potential using the JPCalcWin software (School of Medical Sciences, Sydney, Australia) [6].

In the microelectrode experiments, relative permeability ratios were calculated according to standard methods from the membrane potentials using Goldman-Hodgkin-Katz theory (GHK) [39, 45].

1 $$ \kern0.5em {U}_{\mathrm{A}}-{U}_{\mathrm{B}}\approx -\frac{R\cdotp T}{F}\cdotp \ln \left(\frac{P_{\mathrm{A}}\cdotp {\left[A\right]}_{\mathrm{o}}}{P_{\mathrm{B}}\cdotp {\left[B\right]}_{\mathrm{o}}}\right) $$

Here, *U*_x_ designates the membrane potential in solution *X*, *F* is the Faraday constant, and *T* is the absolute temperature, while *P*_x_ designates the permeability, [*X*]_i_ the inside, and [*X*]_o_ the outside concentration of ion *X*. Note that this equation assumes that the leak current is low as are the contributions of extracellular K^+^ and intracellular Cl^−^.

Single-channel data were analyzed as described previously [23, 48] and in the Supplement. To gain an overview of the conductances determined from the analysis of different patches, data were plotted in amplitude histograms. For this, data from all patches were collected, after which the conductance range was divided into a number of equidistant bins, which were plotted on the horizontal axis. The vertical axis gives the number of patches with a conductance falling into the corresponding bin on the *X*-axis. Note that the bars in these histograms do not correspond to discrete conductance steps.

### Statistical analysis

All data were statistically evaluated using SigmaStat 11.0. After testing for normality using the Shapiro-Wilk test, comparisons between two groups were performed using the Mann-Whitney rank sum test. In cases where different solutions were applied consecutively, differences were evaluated using ANOVA on ranks followed by the Student-Newman-Keuls method for multiple comparisons or the Wilcoxon signed-rank test for pairwise comparisons. A significant difference was assumed if *p* ≤ 0.05.

Obtained values were given as means ± SEM, rounding as recommended by the DIN 1333 [17]. The *n* value represents the amount of individual experiments whereas *n*/*N* refers to the number of experiments (*n*) from different animals (*N*).

## Results

### Immunohistochemical detection of bTRPV3 in the ruminal epithelium

**Fig. 1:**
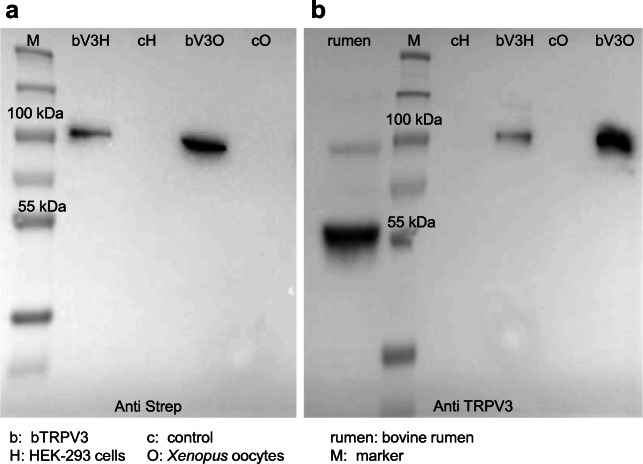
Comparative immunoblots to validate specific binding of the TRPV3 antibody. **a** Identification via Strep antibody: the marker lane (M) is followed by HEK-293 cells transfected with the Strep-tagged bTRPV3 construct (bV3H) or the control vector (cH), followed by oocytes injected with Strep-tagged bTRPV3 (bV3O) or water (cO). A strong band can be seen at ~ 90 kDa in the overexpressing samples but not in controls, representing Strep-tagged bTRPV3. **b** Identification via TRPV3 antibody: the four lanes to the right of the marker lane (M) represent bTRPV3 and controls as in **a**. On the left-hand side, bovine ruminal protein (rumen) has been added, showing a band at about ~ 90 kDa. A second, more prominent band is observed at ~ 60 kDa. (protein loading: HEK-293 0.02 μg, oocytes 0.20 μg, rumen 50 μg)

In a first step, the bovine gene for *TRPV3* was sequenced from ruminal tissue (GenBank: MF063038.1; https://www.ncbi.nlm.nih.gov/nuccore/1220516332). The resulting sequence of 2397 base pairs (bp) showed ~ 90% homology with human *TRPV3*. Via epitope screening, a promising TRPV3 antibody was selected and its suitability controlled in the two expression systems *Xenopus laevis* oocytes and HEK-293 cells. Successful bTRPV3 expression was confirmed by an immunoblot with detection of the Strep-tag (*n* = 8, Fig. 1a), demonstrating a band at the predicted molecular weight of bTRPV3 (~ 90 kDa) in overexpressing cells, but not in controls. In a second step, the TRPV3 antibody was tested in both expression systems and in protein samples from bovine rumen (*n*/*N* = 7/5, Fig. 1b). Both overexpressing and ruminal samples showed a band at ~ 90 kDa. Controls showed no staining. In protein from native ruminal epithelium, the TRPV3 antibody stained a second, stronger band at ~ 60 kDa. The blot was repeated in the absence and presence of the specific peptide (SIP) used to produce the TRPV3 antibody. Co-incubation with SIP markedly reduced staining intensity, proving binding of the TRPV3 antibody via the domains specific for the target epitope (see methods and the Supplement).

**Fig. 2:**
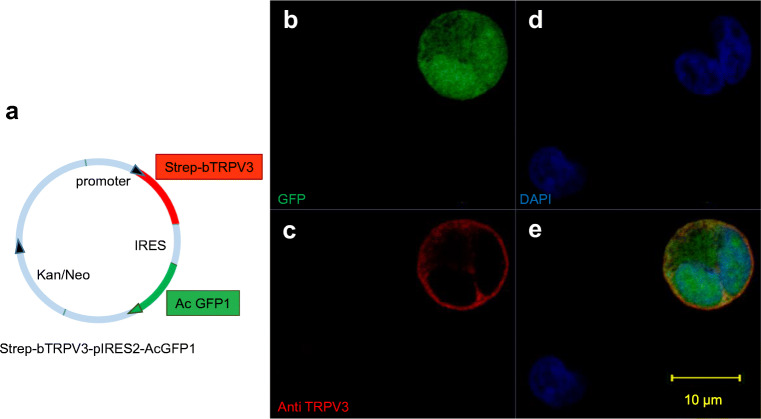
Immunohistological staining of overexpressing bTRPV3 HEK-293 cells. **a** Vector used for transfection. The *bTRPV3* gene is fused to a Strep-tag, but not to green fluorescent protein (GFP). **b** Immunohistological staining reveals successful expression of cytosolic GFP. **c** Staining with the TRPV3 antibody (red) shows expression in the cellular membrane. **d** All cell nuclei were stained with DAPI (blue). **e** Overlay of **b**, **c**, and **d**. The cell in the top right-hand corner is in the process of division with both halves expressing bTRPV3 and GFP. The cell in the lower left-hand corner was not successfully transfected

**Fig. 3:**
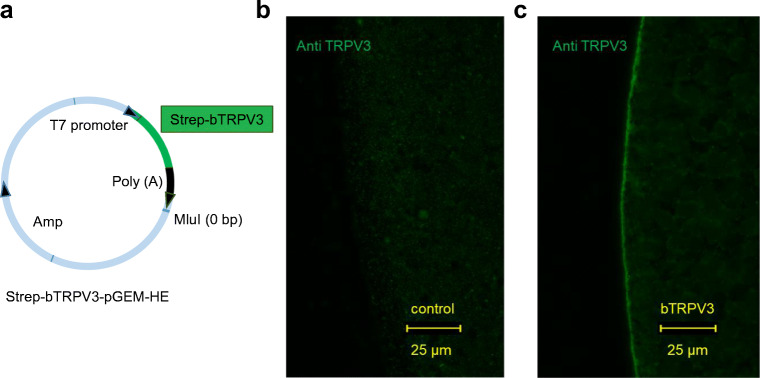
Immunohistological staining of bTRPV3 in *Xenopus* oocytes. **a** pGEM construct containing a Strep-tagged *bTRPV3* sequence for *in vitro* transcription to cRNA. **b** and **c** Immunohistological staining of two oocytes 4 days after injection of **b** water or **c***bTRPV3* cRNA. Cells were stained with TRPV3 antibody and investigated using the same microscope settings. Only overexpressing oocytes **c** show staining of the cellular membrane with the TRPV3 antibody

**Fig. 4:**
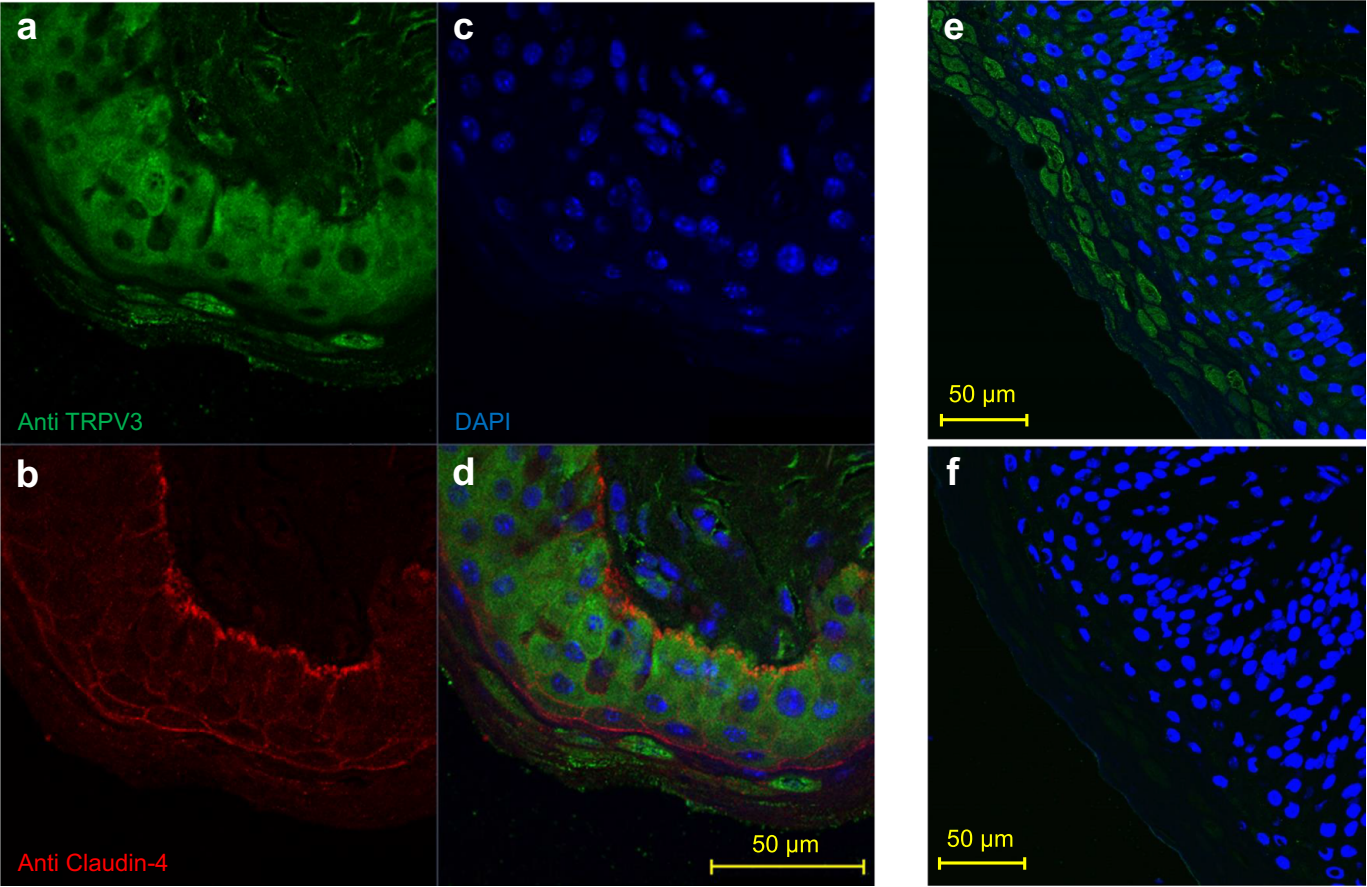
Immunohistological staining of native ruminal epithelium. **a** Staining of ventral rumen with the antibody against bTRPV3 (green), demonstrating strong staining of all epithelial layers. Although staining of peripheral structures (most likely representing the cellular membrane) could be clearly seen, cytosolic staining was intense throughout. **b** Claudin-4 (red) forms junctions between the cells. **c** Cell nuclei are shown in blue (DAPI). **d** Overlay of **a**, **b**, and **c**. **e** Staining of dorsal rumen with the bTRPV3 antibody and DAPI. **f** Using the same rumen sample and same microscope settings as in **e**, treatment with both the TRPV3 antibody and its immunizing peptide (SIP) prevented most of the staining for bTRPV3

For imaging, HEK-293 cells (*n* = 2, Fig. 2) and oocytes (*N* = 3, Fig. 3) were stained with the TRPV3 antibody. In both cases, the cell membrane showed staining for bTRPV3 in overexpressing cells, but not in cells treated with the secondary antibody only or in control cells. Finally, native bovine ruminal tissue from four different animals was stained using the TRPV3 antibody (Fig. 4). Epithelial layers from the *stratum basale* to the *stratum granulosum* were strongly stained, with detection both in the cellular membrane and in the cytosol. In addition, a number of cells within the lower parts of the *stratum corneum* showed staining. Conversely, staining of structures within the subepithelial layers was weak. Treatment with the secondary antibody only did not result in staining. Co-incubation with SIP strongly reduced staining (Fig. 4e, f).

### Experiments with pH-sensitive microelectrodes

Since *Xenopus* oocytes are an established system for studying ammonia transport [12, 24], the intracellular pH (pH_i_) and the membrane potential (*U*_mem_) of oocytes expressing bTRPV3 and controls were investigated using pH-sensitive double-barrelled microelectrodes.

**Fig. 5:**
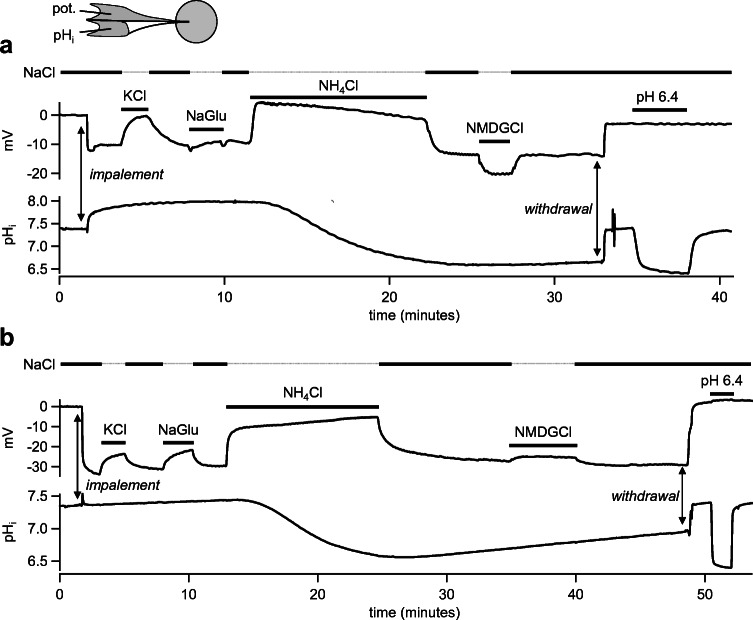
Original recordings of two oocytes expressing bTRPV3 measured via pH-sensitive, double-barreled microelectrodes. All oocytes uniformly responded to KCl and NH_4_Cl with a rapid and reversible depolarization. Only application of NH_4_Cl induced a strong and reversible acidification. Responses to NaGlu and NMDGCl varied, as shown in **a** and **b** and discussed in the text

In a first set of screening experiments on oocytes, the effect of a replacement of bath Na^+^ by K^+^, NH_4_^+^, and NMDG^+^ was investigated, as well as the effect of a replacement of Cl^−^ by gluconate (Glu^−^). All successfully impaled bTRPV3 oocytes responded to the application of KCl solution with a depolarization (from − 20 ± 4 mV to − 12 ± 4 mV, *n*/*N* = 6/1, *p* = 0.006) with recovery to − 18 ± 3 mV (Fig. 5 a and b). Responses to Glu^−^ were variable, with little impact on *U*_mem_ in three of the six oocytes studied (Fig. 5a). The other three oocytes were depolarized by Δ*U*_mem_ = 14 ± 4 mV (Fig. 5b), reflecting expression of Cl^−^ channels [38, 42]. However, overall, effect of Glu^−^ did not pass testing for significance (*p* = 0.6), so that *U*_mem_ appears to be primarily determined by the cation conductances:

2 $$ {U}_{\mathrm{mem}}=\frac{R\cdotp T}{F}\cdotp \ln \left(\frac{P_{\mathrm{Na}}\cdotp {\left[{\mathrm{Na}}^{+}\right]}_{\mathrm{o}}+{P}_{\mathrm{K}}\cdotp {\left[{\mathrm{K}}^{+}\right]}_{\mathrm{o}}}{P_{\mathrm{Na}}\cdotp {\left[{\mathrm{Na}}^{+}\right]}_{\mathrm{i}}+{P}_{\mathrm{K}}\cdotp {\left[{\mathrm{K}}^{+}\right]}_{\mathrm{i}}}\right)\approx \frac{R\cdotp T}{F}\cdotp \ln \left(\frac{P_{\mathrm{Na}}\cdotp {\left[{\mathrm{Na}}^{+}\right]}_{\mathrm{o}}}{P_{\mathrm{K}}\cdotp {\left[{\mathrm{K}}^{+}\right]}_{\mathrm{i}}}\right) $$

Mean pH_i_ was not visibly affected by these solution changes, but rose continuously from 7.39 ± 0.12 after impalement to 7.61 ± 0.10 after washout of Glu^−^ (*p* = 0.07). Subsequent application of NH_4_Cl solution resulted in a rapid depolarization of all bTRPV3 oocytes studied (− 4.0 ± 2.2 mV, *p* = 0.008) with a highly significant pH_i_ decline to 6.22 ± 0.18 (*p* = 0.006). Return to NaCl solution resulted in an immediate repolarization of all oocytes to − 17 ± 3 mV. Intracellular pH recovered more gradually, reaching 6.27 ± 0.14 after 10 min. Replacement of Na^+^ by NMDG^+^ during the recovery phase had no impact on pH_i_, but induced a strong reversible hyperpolarization in three oocytes (Δ*U*_mem_ = 10 ± 4 mV, Fig. 5a), reflecting reduced influx of cations. Unexpectedly, the three other bTRPV3 oocytes responded with a slight depolarization (Δ*U*_mem_ = 2.7 ± 0.3 mV, Fig. 5b). This may reflect induction of membrane leakage by high concentrations of NMDG^+^ as described previously [38]. Due to converse reactions, the overall effect of NMDG^+^ was not significant (*p* = 0.5). In seven control oocytes from the same frog, effects were not significantly different, although interestingly, six of them responded to NMDG^+^ with a hyperpolarization.

**Fig. 6:**
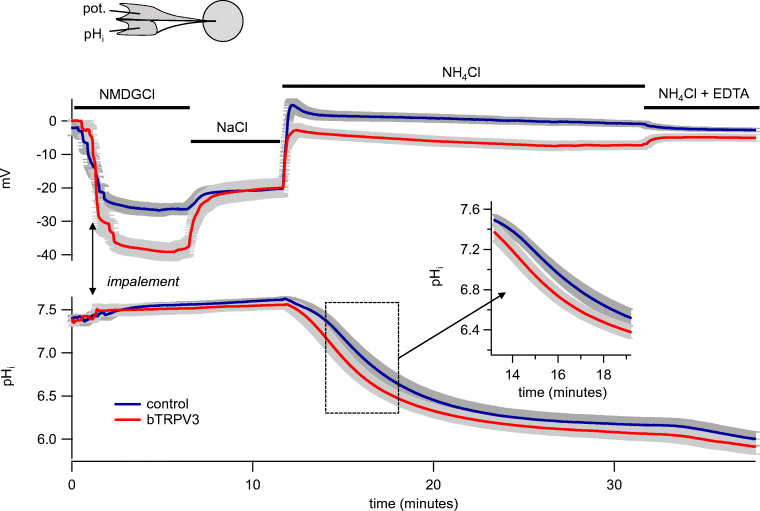
Intracellular pH (pH_i_) and membrane potential (in mV) of oocytes expressing bTRPV3 (*n*/*N* = 14/3) and control oocytes (*n*/*N* = 16/3). The blue traces show the means (± SEM in gray) of all control oocytes, the red traces the means (± SEM in gray) of all bTRPV3 oocytes. Overexpressing bTRPV3 oocytes had a significantly lower membrane potential in NMDGCl than controls, reflecting higher efflux of K^+^ through bTRPV3 channels. Subsequent application of NaCl led to a stronger potential jump in oocytes expressing bTRPV3. The final potential was equal to that of controls, reflecting both a higher influx of Na^+^ and a higher efflux of K^+^. Relative to NMDGCl, NH_4_Cl solution induced a stronger depolarization in bTRPV3 oocytes than in controls, reflecting higher influx of NH_4_^+^. However, the final potential was slightly lower in bTRPV3 oocytes, suggesting a relatively higher efflux of K^+^ in bTRPV3 oocytes. Acidification after application of NH_4_^+^ was significantly faster in bTRPV3 oocytes. Ultimately, both systems reached similar pH_i_. Note the inverse responses in membrane potential after a switch to a divalent cation-free NH_4_Cl solution (EDTA) (details see text and Table 2)

In a second set of experiments, the effect of NH_4_^+^ was studied more rigidly in *Xenopus* oocytes from three animals, strictly alternating between bTRPV3 and control oocytes (Fig. 6 and Table 2). To assess leak currents, experiments started in an NMDGCl solution, in which bTRPV3 oocytes had a significantly lower *U*_mem_ than controls. This undoubtedly reflects higher efflux of K^+^ through bTRPV3 channels, resulting in a lower resting potential. A slight continuous alkaline drift was observed. Replacement of NMDG^+^ by Na^+^ did not change this drift in pH_i_, suggesting that any baseline activity of NHE was discrete. Addition of Na^+^ resulted in a strong depolarization of all oocytes studied, with a potential jump significantly higher in the bTRPV3 group, but reaching the same end level as in the controls. According to GHK theory, this is to be expected if overexpression of bTRPV3 increases both the permeability to K^+^ (i.e., K^+^ efflux) and the permeability to Na^+^ (i.e., Na^+^ influx) by the same factor. While absolute currents should increase, the *U*_mem_ (Eq. 2) should remain roughly the same.

**Table 2:** Membrane potential, intracellular pH and relative permeability ratios of bTRPV3 expressing (bV3, *n*/*N* = 14/3) and control *Xenopus* oocytes (ctrl, *n*/*N* = 16/3). Solutions were applied consecutively and values were measured 5 min after exposure unless indicated otherwise. Relative permeability ratios were calculated from Eq. 1 and contain contributions of leak currents. The last column gives the *p* values for differences between the two columns. Within columns, different superscripts indicate significant differences with *p* < 0.05

Membrane potential (mV)			
	bTRPV3	control	p (bV3 vs. ctrl)
NMDGCl	-39.2 ± 2.6^a^	-27.2 ± 1.8^a^	0.001
NaCl	-20.1 ± 2.8^b^	-20.3 ± 2.2^b^	0.6
NH_4_Cl (3.5 min)	-4.4 ± 1.5^d^	1.5 ± 1.4^c^	0.003
NH_4_Cl (20 min)	-7.2 ± 1.3^c^	-0.9 ± 0.8^d^	≤0.001
NH_4_Cl-EDTA	-5.0 ± 1.1^d^	-2.7 ± 0.5^e^	0.03
Intracellular pH			
	bTRPV3	control	p (bV3 vs. ctrl)
NMDGCl	7.52 ± 0.07^a^	7.56 ± 0.04^a^	1.0
NaCl	7.56 ± 0.05^b^	7.61 ± 0.03^b^	0.9
NH_4_Cl (3.5 min)	6.90 ± 0.08^c^	7.14 ± 0.06^c^	0.04
NH_4_Cl (20 min)	6.08 ± 0.08^d^	6.18 ± 0.09^d^	0.6
NH_4_Cl-EDTA	5.96 ± 0.09^e^	6.05 ± 0.08^e^	0.6
Relative permeability ratio p(X) / p(NMDG^+^)			
Ion X	bTRPV3	control	p (bV3 vs. ctrl)
Na^+^	2.16 ± 0.19^a^	1.35 ± 0.11^a^	0.002
NH_4_^+^ (20 min)	3.41 ± 0.22^b^	2.78 ± 0.21^b^	0.06
NH_4_^+^ (EDTA)	3.76 ± 0.30^c^	2.58 ± 0.17^c^	0.003

Application of NH_4_^+^ caused a further depolarization in both cell types. Relative to the potential in NMDGCl, the magnitude of depolarization was significantly higher in oocytes expressing bTRPV3, suggesting higher influx of NH_4_^+^. However, in absolute terms, the resulting *U*_mem_ in NH_4_^+^ was lower in bTRPV3 expressing oocytes, again reflecting higher K^+^ efflux. From these potentials, relative permeability ratios can be determined that are given in Table 2, which showed that relative to NMDG^+^, bTRPV3 expressing oocytes showed significantly higher conductances to both Na^+^ and NH_4_^+^. In line with this, after application of NH_4_^+^, an acidification was observed in all oocytes that was significantly faster in bTRPV3 oocytes than in controls, suggesting a higher absolute influx of NH_4_^+^ ions. However, final pH_i_ did not differ, possibly reflecting an equilibrium primarily determined by the potential, the concentration gradients, and the pH regulatory mechanisms rather than by the permeability.

Subsequent removal of Ca^2+^ from the solution with buffering by EDTA had inverse effects on bTPRV3 expressing and control oocytes. As expected, all bTRPV3 oocytes were depolarized by removal of Ca^2+^ in line with greater influx of NH_4_^+^ through bTRPV3 after removal of Ca^2+^, which permeates the pore of bTRPV3 with high affinity, thus interfering with the entry of monovalent cations. More surprising was the observation that all control oocytes were hyperpolarized. Most likely, this reflects a lower expression of divalent-sensitive cation channels in conjunction with an opening of calcium-inactivated Cl^−^ channels that are endogenously expressed by *Xenopus* oocytes [42]. In both groups of oocytes, application of EDTA resulted in a further slight acidification. In bTRPV3 oocytes, speed of acidification rose from (− 8 ± 4) 10^−3^ pH units/min before application of EDTA to (− 34 ± 6) 10^−3^ pH units/min (*p* ≤ 0.001) 5 min later, in controls, from (− 3.2 ± 1.7) 10^−3^ pH units/min to (− 41 ± 9) 10^−3^ pH units/min (*p* = 0.006), with no significant difference between the groups (*p* = 0.11). Likewise, the end value of pH_i_ did not differ between the two groups.

In conjunction, these results suggest that both groups of oocytes express conductances to K^+^, Na^+^, and NH_4_^+^, with permeability relative to NMDG^+^ (plus leak currents) higher in bTRPV3 oocytes (Table 2). In both groups, any permeability to NH_3_ is much smaller than that to NH_4_^+^.

### Patch-clamp experiments

Subsequent inside-out patch-clamp experiments on membrane patches of oocytes confirmed this hypothesis, with both groups showing a conductance to NH_4_^+^ on the single-channel level. In total, patches from 27 bTRPV3 and 21 control oocytes from the same three frogs used in microelectrode experiments were investigated in the inside-out configuration, alternating between the two groups. To allow an assessment of the NH_4_^+^ conductance under symmetrical conditions, the experiments were carried out with NH_4_^+^ in the pipette.

Throughout and in both groups, there was a tendency for single-channel events to occur in one solution and vanish in another solution without apparent reason. Typically, activity of channels increased with the duration of the measurement. Patches were frequently silent in the first solution (NaCl), and showed activity after application of the second solution (NH_4_Cl). Those patches that survived a return to NaCl remained active, arguing against a selective conductance to NH_4_^+^.

**Fig. 7:**
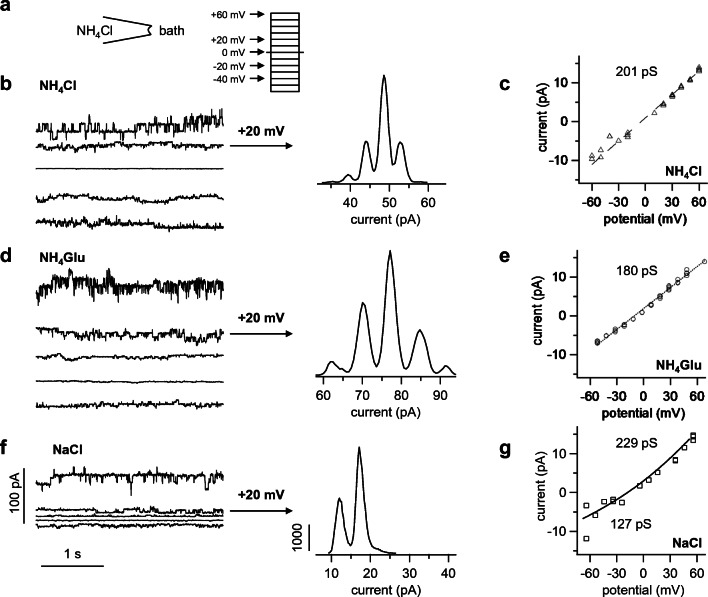
Single-channel measurements from a bTRPV3 expressing oocyte (inside-out). **a** Measurements were performed with NH_4_Cl in the pipette. The cytosolic side of the patch was consecutively exposed to different bath solutions as indicated and exposed to potentials between − 60 and + 60 mV in steps of 10 mV. For clarity, only the current responses to the potentials indicated by the arrows are shown. **b** Original recording in NH_4_Cl bath solution (same scaling as in **f**). The amplitude histogram at + 20 mV can be seen in the middle showing three distinct channels. **c** IV-plot corresponding to **b**, yielding a linear relationship with the slope of the fit as indicated in the figure. **d** Channel openings were not affected by the replacement of chloride by the much larger anion gluconate, proving cation selectivity (same scaling as in **a** and **f**). The histogram in the middle shows four distinct channels. **e** IV-plot from **d**, with the GHK fit yielding a similar conductance as in **c**. **f** In NaCl solution, channel openings at positive potentials are comparable with **b** and **d**, whereas channel openings at negative potentials were smaller. **g** IV-plot from **f**, fitted with the GHK equation by variation of the permeability to the two ions Na^+^ and NH_4_^+^. The conductance was then calculated from the GHK fit to the data and from the concentrations (see Supplement, equation 2). The negative reversal potential of the fit reflects a higher conductance to NH_4_^+^

Of the 27 patches from bTRPV3 expressing oocytes, all but four showed channel activity in at least one trace (Fig. 7). The conductance could be determined using linear fits for symmetrical (Fig. 7 c and e) or the GHK equation for asymmetrical configurations (Fig. 7g, see methods). Ion replacement showed that the conductance depended on the cation, but not on the anion in the bath. Over all patches, single-channel conductance to NH_4_^+^ in symmetrical solution was 92 ± 15 pS, (*n* = 18).

**Fig. 8:**
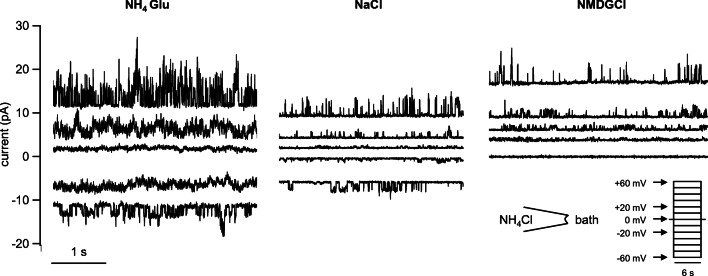
Original recordings from an inside-out patch from a control oocyte (same scaling). Measurements were performed with NH_4_Cl in the pipette. Only the current responses to the potentials indicated by the arrows are shown in the traces. Measurement in NH_4_Glu solution showed small channel openings at positive and negative potentials, with the fit yielding a conductance of 41 pS for NH_4_^+^ in this patch. After switching to NaCl solution, channel openings at negative potentials were visibly smaller, reflecting influx of Na^+^ (here: conductance of 59 pS for NH_4_^+^ and 31 pS for Na^+^). After replacement of Na^+^ with the much larger cation NMDG^+^, channel openings were only visible at positive potentials. The GHK fit yielded a conductance for NH_4_^+^ of 53 pS and 11 pS for NMDG^+^

In control oocytes, only 14 out of 21 patches showed channel activity in at least one solution (Fig. 8). In asymmetrical solution with NaCl in the bath, the conductance of these small channels was about half of those found in bTRPV3 oocytes (48 ± 9 pS, *n* = 11, *p* = 0.04). An almost identical NH_4_^+^ conductance was determined from linear fits after switching to symmetrical NH_4_Cl solution (43 ± 9 pS, *n* = 13, *p* = 0.7). In four patches, it was possible to switch to KCl and NMDGCl solutions, yielding similar values for NH_4_^+^ (*p* = 0.7). The conductances to the other ions Na^+^ (33 ± 10 pS, *n* = 11, *p* = 0.11), K^+^ (23.8 ± 1.3 pS, *n* = 2, *p* = 0.4), and NMDG^+^ (9.2 ± 2.1 pS, *n* = 3, *p* = 0.10) were numerically smaller than the NH_4_^+^ conductance.

**Fig. 9:**
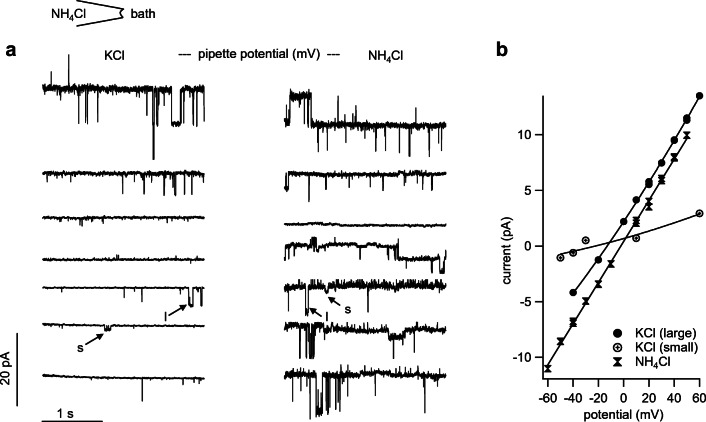
Inside-out measurement of a patch from a bTRPV3 oocyte expressing two different types of channels. The pipette was filled with NH_4_Cl. **a** Original recordings, showing consecutive exposure to KCl (traces to the left) and NH_4_Cl (traces to the right, same scaling). Small (s) and large (l) populations of channels were observed, most likely representing endogenous and bTRPV3 channels respectively. **b** IV-plot of unitary currents from amplitude histograms of the patch in **a**. Data from the symmetrical NH_4_Cl configuration were fitted linearly and yielded a conductance of 185 pS, with a reversal potential ~ 0 mV. In the asymmetrical KCl configuration, data from large and small channels were fitted separately to the GHK equation by variation of the permeability to the two ions NH_4_^+^ and K^+^. The conductance was calculated from the permeability and the concentrations (see Supplement, equation 2). The fit of the large channel openings yielded a conductance to NH_4_^+^ of 215 pS and to K^+^ of 129 pS. The smaller openings could be fitted with a conductance of 45 pS for NH_4_^+^ and 18 pS for K^+^. Both reversal potentials were shifted to ~ − 15 mV, confirming the higher conductance to NH_4_^+^

Compared with the control oocytes, the channel population in patches from bTRPV3 oocytes was visibly much more diverse. While 14 patches showed large conductances similar to those in Fig. 7, nine other patches showed smaller channels comparable with those seen in the control oocytes. In eight patches, both small and large channels could be observed in the same experiment and three of them simultaneously showed large and small channel activity in the same trace. The conductance of bTRPV3 for K^+^ could be investigated in two of such patches (Fig. 9). The large channels in these patches had a conductance of 216 ± 2 pS for NH_4_^+^ and 116 ± 13 pS for K^+^ and most likely reflected bTRPV3 channels. The smaller conductances of 52 ± 11 pS for NH_4_^+^ and of 43 ± 14 pS for K^+^ (*n* = 5) appeared to reflect endogenous channels.

**Fig. 10:**
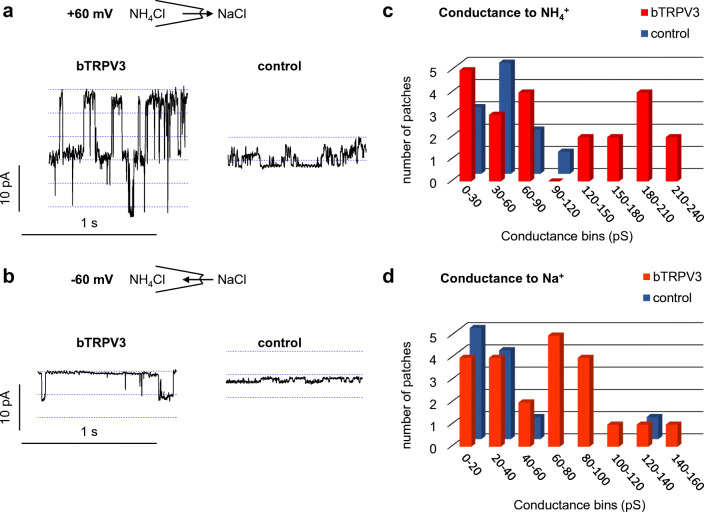
Original inside-out recordings and histograms from control and bTRPV3 oocytes in asymmetrical solution with NH_4_Cl in the pipette and NaCl in the bath. **a** Original recordings from one overexpressing bTRPV3 oocyte and one control oocyte at a pipette potential of + 60 mV, reflecting efflux of NH_4_^+^ (same scaling). **b** Corresponding traces at − 60 mV, reflecting Na^+^ influx. Data from all voltages were fitted as in Figs. 7 and 8 to yield a conductance to NH_4_^+^ and Na^+^ for each patch. **c** Histogram giving an overview of all conductance values for NH_4_^+^ determined from patches showing channel activity in asymmetrical solution. The total conductance range was divided into a number of equidistant bins, which are given on the *X*-axis. The *Y*-axis gives the number of patches with a conductance falling into the corresponding bin on the *X*-axis. The histogram shows one cluster of NH_4_^+^ conductances for control oocytes (blue) around 50 pS, while for bTRPV3 oocytes (red), a second cluster of conductances can be seen around 150 pS. Three bTRPV3 patches expressed both small and large channels. **d** Corresponding histogram of all measurements of the conductance to Na^+^. One peak emerges at ~ 20 pS for both groups of oocytes and a second peak at ~ 80 pS in bTRPV3 oocytes only

Despite some overlap, the two populations of channels become apparent in the amplitude histogram of NH_4_^+^ conductances (Fig. 10c). The vertical height of the bars represents the number of patches falling into a certain conductance range, which is given on the horizontal axis. While control oocytes had one peak at conductances around 50 pS, oocytes expressing bTRPV3 showed a second peak over 150 pS. In the corresponding histogram for Na^+^ conductances, a similar distribution was observed with one peak at ~ 20 pS for both groups and a second peak at ~ 80 pS in bTRPV3 oocytes only (Fig. 10d). In the further analysis, we assumed that channel activity smaller than 100 pS for NH_4_^+^ reflects expression of endogenous non-selective channels, which were excluded from subsequent statistical evaluation. Under these circumstances, the mean NH_4_^+^ conductances from these traces were 144 ± 12 pS (*n* = 9) for symmetrical NH_4_Cl solution, 185 ± 14 pS (*n* = 6, *p* = 0.7 vs. NH_4_Cl) for the NH_4_Glu bath solution, and 182 ± 12 pS (*n* = 10, *p* = 0.04 vs. NH_4_Cl) for the NaCl bath solution. The latter also yielded a conductance to Na^+^ (98 ± 10 pS), significantly lower than that for NH_4_^+^ (*p* ≤ 0.001).

The current study was performed in modifications of oocyte Ringer solution and accordingly, the conductances differed from those obtained in mammalian Ringer solution. However, after adjusting for the different concentrations of NH_4_^+^ (Supplement, equation 3), the conductance of the large channels in the bTRPV3 group could be statistical compared with the data which we obtained in a previous study of bTRPV3 expressed in HEK-293 cells [48]. For symmetrical NH_4_Cl solution, no significant differences were found (*p* = 0.8). In asymmetrical solution, there was a slight trend for a lower NH_4_^+^ conductance in oocytes (*p* = 0.08). The Na^+^ conductance did not differ (*p* = 0.9).

## Discussion

**Fig. 11:**
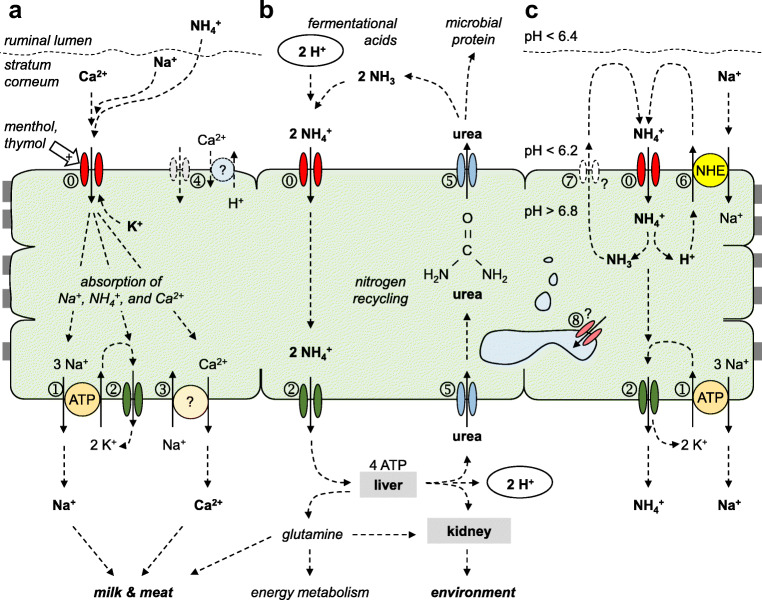
Model showing the function of bTRPV3 in the rumen. The ruminal epithelium is a multilayered, *squamous* epithelium of cells that are interconnected by gap junctions, thus forming a functional syncytium. **a** bTRPV3 (⓪) is a non-selective cation channel that can serve as a pathway for the uptake of nutrients such as Na^+^ and Ca^2+^, and contributes to the apical conductance for K^+^. Uptake of cations is stimulated by certain monoterpenoids such as menthol and thymol. Basolateral extrusion involves the sodium-potassium pump (ATP1A1,➀), basolateral K^+^ channels (➁), and sodium-calcium exchangers (➂). In the model, NH_4_^+^ is taken up by the same pathway as K^+^ (⓪,➁). Other TRP channels and exchangers may be involved (➃). **b** Within the ruminal lumen, large quantities of fermentational acids are produced, releasing protons that can partially be removed via efflux of NH_4_^+^ via bTRPV3 (⓪) and basolateral K^+^ channels (➁). In the liver, NH_4_^+^ is converted to non-toxic metabolites, mostly urea, but also some glutamine. Only glutamine can be utilized by mammalian enzymes for protein synthesis. Conversely, urea must be excreted. This can occur renally, resulting in nitrogen losses and environmental damage. Alternately, urea can be secreted into the rumen via urea transporters such as UT-B or aquaporin 3 (➄). After degradation by the microbiota within, NH_3_ is released and can be utilized by microbial enzymes for protein synthesis. NH_3_ also functions as a buffer, binding protons to form NH_4_^+^ that is again removed via bTRPV3 (⓪). This “nitrogen recycling” can reach 20 mol day^−1^ in cattle. **c** At physiological pH gradients across the apical membrane, NH_4_^+^ stimulates sodium transport via NHE (SLC9A3,➅) with apical recirculation of NH_3_ via an unknown pathway (➆). Electrogenic transport of NH_4_^+^ across the basolateral membrane continues (➁). Specific staining for bTRPV3 can also be found within the cytosol, possibly reflecting expression of bTRPV3 or its splice variant in intracellular membranes such as those of the ER (➇)

The current study provides clear evidence for expression of the bovine homologue of TRPV3 by the epithelial layers of the bovine rumen. We confirmed the permeability of this channel to NH_4_^+^, Na^+^, and K^+^ in Ringer solutions with physiological concentrations of Ca^2+^ and Mg^2+^. In conjunction with previous studies of our group [8, 40, 45, 48], we conclude that bTRPV3 is involved in the ruminal uptake of NH_4_^+^, Na^+^, and Ca^2+^ and contributes to the apical conductance of K^+^ [29] (Fig. 11).

In a first step, the bovine *TRPV3* was sequenced. After epitope screening, a commercial murine antibody was selected with binding affinity to a conserved epitope in the first extracellular loop of TRPV3. In immunoblots of protein from overexpressing HEK-293 and *Xenopus* oocytes, the antibody stained a band at the predicted height of ~ 90 kDa (Fig. 1). In non-expressing controls from both groups, no band was observed proving that staining was caused by binding of the antibody to bTRPV3. Corresponding immunohistochemistry confirmed expression of the channel protein primarily in the cellular membrane (Figs. 2 and 3). Immunoblots of ruminal protein show a band of equivalent molecular weight (~ 90 kDa), clearly arguing for expression of bTRPV3 by the rumen. However, a second, stronger band was visible at ~ 60 kDa (Fig. 1b). In a previous study, a ~ 60 kDa band could also be observed in human epidermal keratinocytes stained with a goat-polyclonal antibody directed against the epitope AA 461-487 of TRPV3 [53]. In that study, knockdown of TRPV3 showed that the ~ 60 kDa band was a splice variant. Blasting the epitope sequence with alignment in NCBI yields only three splice variants of bTRPV3, two at ~ 90 kDa, and only one with a predicted length of 60.25 kDa (NP_001092494.1). This splice variant contains the epitope for binding of the antibody and thus appears as the most likely explanation for the additional ~ 60 kDa band in Fig. 1b.

In particular in immunohistochemical staining, antibodies may occasionally bind by their constant domains rather than via the high affinity binding domains for the target epitope [9]. For this reason, immunochemical stainings and immunoblots were repeated in presence of the peptide used to produce the TRPV3 antibody. If the antibody is functional, this peptide should interfere with specific binding via the high affinity binding domains. Non-specific binding via other domains should continue unimpaired. In our case, a quenching of staining was observed (Fig. 4 f and Supplement). However, it should be emphasized that ultimately, this observation does not rule out that binding might have occurred to another protein via a structure similar to the target epitope [9].

*TRPV3* emerged as a candidate gene when searching for the apical divalent-sensitive, non-selective cation channel of the rumen [30, 45, 49, 59]. Traditionally, TRP channels have been primarily regarded as channels involved in neuronal signaling rather than in epithelial transport. However, both spontaneous and induced mutations of TRPV3 primarily interfere with the function of keratinocytes, inducing skin lesions rather than neurological symptoms [36]. In line with this, ruminal tissue showed a strong immunohistochemical staining of bTRPV3 within the epithelium while subepithelial staining was weak (Fig. 4). Staining was observed not only in the cellular membranes of the ruminal epithelium, but also in the cytosol. This is in line with findings in human keratinocytes or intact epidermis, where similar staining patterns were found [53].

Research in recent years has established that almost all TRP channels studied so far are expressed not only by the plasma membrane as previously thought, but also by intracellular vesicular membranes [19]. Thus, a recent study of the skin suggests expression of TRPV3 by the endoplasmic reticulum (ER) [64] and an involvement of TRPV3 in lysosomal function. It should also be noted that in the course of cell differentiation in stratified squamous epithelia, granules dissociate from the ER via blebbing and grow in size until they become visible as the keratohyalin granules that give the *stratum granulosum* its name [21]. Here, the proteins needed for keratinization are produced, requiring high quantities of glutamine. Accordingly, glutamine synthetase is highly expressed by the skin [16] and the rumen [47]. Within the ER, this enzyme catalyzes formation of glutamine from NH_4_^+^, possibly requiring additional uptake routes from the cytosolic space. TRPV3 would certainly fulfill this role (Fig. 11c).

However this may be, there is convincing evidence to suggest that bTRPV3 mediates transport of NH_4_^+^ across the ruminal epithelium. In Ussing chamber experiments, TRPV3 channel agonists stimulate currents carried by Na^+^ and NH_4_^+^ and enhance Ca^2+^ flux [40, 45] (Fig. 11a). Furthermore, at physiological ruminal pH, exposure to NH_4_^+^ acidifies the cytosolic space of native ruminal epithelia from sheep and cattle as measured by pH-sensitive microelectrodes [32, 45]. Flux measurements confirm concomitant stimulation of NHE [3] (Fig. 11c). This has profound implications for the role of this channel in maintaining the pH of the ruminal fluid (Fig. 11b). As mentioned, large quantities of urea are secreted by the rumen and degraded according to:

3 $$ {\left(\mathrm{N}{\mathrm{H}}_2\right)}_2\mathrm{CO}+{\mathrm{H}}_2\mathrm{O}\to 2\ \mathrm{N}{\mathrm{H}}_3+\mathrm{C}{\mathrm{O}}_2 $$

At physiological ruminal pH of 6.4, over 99.9% of NH_3_ is immediately converted to NH_4_^+^. Absorption of NH_4_^+^ via TRPV3 results in a permanent removal of these protons from the rumen. In cattle on high-energy diets with production of large quantities of fermentational acids, influx of urea and efflux of NH_4_^+^ via bTRPV3 might represent an essential mechanism for ruminal pH homeostasis [5], explaining the high levels of nitrogen recycling observed in these animals, and the large quantities of ammonia that they excrete into the environment (Fig. 11b).

The current study confirms that bTPRV3 can serve as a pathway for protons bound in the form of NH_4_^+^. Microelectrode studies show a strong acidification of the cytosol of *Xenopus* oocytes expressing bTRPV3 (Fig. 5). Although NH_4_Cl generally leads to an increase of the pH_i_ via influx of NH_3_, an acidification has been observed in a number of systems such as astrocytes [34] or the ruminal epithelium [32, 45] and is generally attributed to influx of NH_4_^+^ with subsequent dissociation to H^+^ and NH_3_ [11] (Fig. 11c). Depending on the pH gradients present across the cellular membrane, NH_3_ can diffuse back into the extracellular space [34], or accumulate in subcellular organelles where it can be detoxified [11]. In our study, we were tempted to calculate the relative permeability ratio of NH_4_^+^ vs. NH_3_ from the equilibrium pH_i_ reached in steady state. In principle, this should be possible but would require knowledge of the distribution of ammonia inside and outside of the cell (Eq. 15 in [44] and Fig. 8 in [34]). Furthermore, the system is not in equilibrium but must have ill-defined mechanisms for pH regulation and detoxification of ammonia, further complicating matters.

It should be stressed that control oocytes were also strongly acidified by application of NH_4_^+^, albeit somewhat less rapidly than in oocytes expressing bTPV3 (Fig. 6). Given that *Xenopus* oocytes have been widely used as expression systems for ammonia-transporting proteins such as Rh-like proteins [12, 24], we did not anticipate the magnitude of the response. It has been noted previously that the lack of a robust expression system for functional analysis of ammonia transport has generally hampered research in the area [35]. Burckhardt and Frömter [11] were among the first to report transport of NH_4_^+^ by native *Xenopus* oocytes. In a careful study using pH-sensitive microelectrodes, the authors showed that endogenously expressed non-selective cation channels were involved. Our single-channel patch-clamp measurements confirm the findings of these authors [11] and clearly demonstrate that native *Xenopus* oocytes robustly express channels that are permeable not only to K^+^ and Na^+^, but also to NH_4_^+^. However, it should also be stressed that these endogenous channels were distinct from the larger channels that were only observed in bTRPV3 overexpressing cells. The conductance values from both groups of channels showed considerable scatter. This certainly reflects both stochastic effects and imprecisions involved with the evaluation and fitting of single-channel data. However, this finding may also reflect the formation of heteromeric channels consisting of bTRPV3 subunits and subunits of smaller endogenous *Xenopus* channels, leading to intermediate conductance levels as previously reported for TRPV1 [14, 15].

In summary, we provide clear evidence for the expression of bTRPV3 by the ruminal epithelium. In conjunction with previous functional studies of the ruminal epithelium [3, 40, 45], a role of this channel in mediating the ruminal transfer of NH_4_^+^ can be assumed. The finding that complex proteins are required to mediate ruminal transport not only of urea [50, 65] but also of ammonium across the ruminal wall should end the concept of ruminal nitrogen recycling as the result of a “leaky” epithelium. Instead, nitrogen recycling appears an efficient mechanism to remove protons from the rumen with the energy coming from the liver (Fig. 11b). Given the robust evidence supporting transport of Ca^2+^ by TRPV3 [37, 45, 48, 63], bTRPV3 also clearly emerges as a candidate mediating electrogenic Ca^2+^ transport by the ruminal epithelium [27, 61, 62].

Certainly, bTRPV3 is not the only NH_4_^+^ transporting channel and there is good reason to believe that in the rumen, in *Xenopus* oocytes, and in other parts of the gut, multiple types of non-selective cation channels should be considered when searching for pathways for the uptake of ammonium [58].

## Electronic supplementary material

ESM 1 (PDF 409 kb)
