# Supplementary material for: The TRPV3 channel of the bovine rumen: localization and functional characterization of a protein relevant for ruminal ammonia transport
Source: Pflugers Arch. 2020 May 26;472(6):693–710. doi: 10.1007/s00424-020-02393-2 (PMC7293678; doi:10.1007/s00424-020-02393-2)
Supplement: Supplementary file 1 — (PDF 409 kb) [file 424_2020_2393_MOESM1_ESM.pdf]

Supplementary Material

The TRPV3 channel of the bovine rumen - localization and functional characterization of a protein relevant for ruminal ammonia transport

Pflügers Archiv – European Journal of Physiology

Franziska Liebe[1*], Hendrik Liebe[1,2*], Sabine Kaessmeyer[3], Gerhard Sponder[1], Friederike Stumpff[1]

email: stumpff@zedat.fu-berlin.de

[1]Institute of Veterinary Physiology, Freie Universität Berlin, Berlin, Germany
[2]Department of Biology, Chemistry, and Pharmacy, Freie Universität Berlin, Germany
[3]Institute of Veterinary Anatomy, Freie Universität Berlin, Berlin, Germany

*) both authors contributed equally to this study

Methods (Supplement)

Immunohistochemical staining

Xenopus laevis oocytes were fixed in paraformaldehyde (4 %, 3 h), washed with PBS (2 times), and dehydrated in increasing concentrations of ethanol (2 times in 70 % (1 h), 70 % (overnight), 3 times in 80 % (1 h), 80 % (72 h), 2 times in 96 % (10 min), 3 times in 99.9 % (10 min)). Xylene was used as intermedium (2 times for 10 min) and finally, oocytes were embedded in paraffin (2 times for 30 min).

Frozen bovine tissue was defrosted in methanol (-20 °C, 1 h). Afterwards, tissue was fixated in formaldehyde (4 % in PBS, 4 °C, 24 h) and dehydrated in increasing ethanol concentrations as follows: 70 % (4 times, each 1 h; last overnight), 80 % (3 times, each 1 h; last overnight), then 80 % (once, 24 h), 96 % (2 times, each 45 min), 99.9 % (3 times, 45 min), methyl benzoate (2 h). Fresh bovine tissues were fixed immediately in formaldehyde and dehydrated with a shorter protocol (70 %, 80 %, 96 %, 99.9 % for 50 min).

For all bovine samples, Xylene was used as intermedium (2x 30 min). The samples were then infiltrated overnight in the incubator at 59°C in paraffin I, afterwards for 2.5h each in paraffin II and III; and finally the samples were embedded in fresh paraffin and

stored in the refrigerator. Slices of 5 µm were cut and mounted on glass slides (Superfrost® Plus Menzel-Gläser, Carl Roth GmbH & Co. KG, Karlsruhe, Germany).

Deparaffinization of cuts of ruminal tissues or oocytes was performed in Roti®-Histol (Carl Roth, Karlsruhe, Germany) overnight. Rehydration was completed by incubating the slides in descending ethanol concentration (99.9 %, 96 %, 90 %, 80 %, 70 %, demineralized water) for 5 minutes respectively.

The slides were washed in PBS (5 min) and boiled in EDTA buffer (1 $mmol\cdot L^{-1}$; oocytes: pH 8.0 adjusted with NaOH; rumen: pH 9.0 adjusted with Tris) for 15 min. Afterwards, the slides were rinsed thrice with PBS (for 1 min and 2 times for 5 min).

HEK-293 cells were seeded onto coverslips, transfected, washed in PBS (2 times), incubated in Roti®-Histofix (4 %, 30 min), and washed again twice in PBS.

Permeabilization of all fixed samples was achieved via incubation in Triton X-100 (0.5 %, 5 min; Merck KGaA, Darmstadt, Germany). After two washing steps with PBS, followed by incubation in blocking solution (BS, goat serum (5 %; PAN-Biotech GmbH, Aidenbach, Germany) in PBS) in a closed container with humidified atmosphere for 1 hour. Samples were then stained with primary antibody diluted in BS (4 °C, overnight) according to Table 1. Secondary antibody controls were performed with BS only.

To test for binding to the epitope, adjacent slices from the same sample of ruminal tissue were incubated in parallel either with the primary mouse TRPV3 antibody (AB_{V3}) only or with a mix of this antibody and its corresponding specific immunizing peptide (SIP) (Table 1).

According to Table 1, samples and secondary antibody controls were incubated in diluted secondary antibodies (in BS supplemented with 4′, 6-diamidine-2′-phenylindole dihydrochloride (1 µg/mL; DAPI, Roche, Mannheim, Germany), 37 °C, 1 h). Slices were washed with BS (2 times for 5 min), demineralized water, and ethanol, followed by embedding (Mount Flour, Biocyc GmbH & Co. KG, Potsdam, Germany) and covering.

Double-barrelled pH-sensitive microelectrode measurements

pH-sensitive microelectrodes were prepared essentially as described previously [1] with a few alterations. For pH-measurements, SUTTER BF 150-86-10 glass tubing (Science Products GmbH, Hofheim, Germany) was used. The reference barrel was made of filamented bisected GC150F 15 glass tubing (Harvard Apparatus, Kent, UK). Two core cable ends (4×10, 611889, Conrad Bauelemente, Conrad Elektronik, Hirschau, Germany) were pushed into tightly fitting shrink tubing (Ø 3 mm) and used to adjoin the two barrels, leaving the middle section free (~ 3 cm). A small piece of shrink tubing (1 mm; Deray-H-set 1/8″, DSG-Canusa, Meckenheim, Germany) was slipped over the end of the reference barrel, slightly separating the barrels at one end. After baking (180°C, 10 min), the piggyback electrodes were pulled with a programmable multipipette puller (PMP-107, Microdata Instrument, South Plainfield, NJ, USA) to give a resistance of ~ 50 MΩ (measured with EPC 9, Heka Elektronik, Lambrecht, Germany). Subsequently, the reference barrel was perfused with pressurized dry air via plastic tubing pushed over one end (~ 0.9 bar). The pH-sensitive barrel was pushed into a rubber insert in the lid of a heated glass jar and exposed to the vapour formed by a drop (200 µl) of fresh dichlorodimethylsilane (Sigma-Aldrich) for 30 min. Pipettes were then baked at 180 °C for 2 h. The pH-sensitive barrel was filled with Hydrogen Ionophore I-Cocktail A (0.2 µl; Sigma Aldrich, St. Louis, MO, USA) via a Microliter syringe (type 7000.50C, Hamilton Company, Reno, NV, USA). The electrodes could be stored in a plastic container with silica gel (P077.2, Carl Roth) and light protection for many months. Once opened, Dichlorodimethylsilane had to be replaced frequently.

On the experimental day, the reference barrel was filled with KCl solution (0.5 $mol \cdot L^{-1}$) via a MicroFil micropipette (34Gauge/67 mm, World Precision Instruments, Sarasota, FL, USA), while the pH-sensitive barrel was filled with KCl/HEPES-buffer (in $mmol \cdot L^{-1}$: 500 KCl, 20 HEPES, pH 7.2 adjusted with Tris). Microelectrodes were then trimmed (0.5-30 MΩ) to remove clogging silane residues using a beveller (BV-10, Sutter Instrument, Novato, CA, USA).

Measurements were performed in a continuously perfused bath chamber. Via chlorinated silver wires, the two barrels were connected to an amplifier (F-223 A Dual Electrometer, World Precision Instruments). The bath was grounded to a common

technical earth via a chlorinated silver wire. In addition, the bath was connected to a commercial electrode (Metrohm, Filderstadt, Germany) via a KCl (3 mol·L^{-1}) agar bridge to minimize liquid junction potential effects (ground signal) [2]. All three signals were measured versus the technical earth and recorded using LabChart 7 software (ADintruments Ltd, Oxford, UK). The potential difference between the two barrels was used to determine the intracellular pH (pH_i), while the potential difference between the reference barrel and the ground signal from the bath corresponded to the membrane potential (U_{mem}).

Electrodes were calibrated before and after each measurement using solution NaCl (pH 7.4) and NaCl-6.4 (pH 6.4). Suitable electrodes showed a stable potential difference of 45 mV or higher measured by the pH-sensitive barrel with effects at the reference electrode < 0.3 mV.

A micromanipulator (Mini 25, Luigs & Neumann, Ratingen, Germany) was used to insert the microelectrode into the oocyte. Impalement was considered to be successful if a sharp drop in the potential across the reference channel to values under -10 mV could be observed.

Analysis of single-channel data

For analysis of single-channel data, an Igor macro (Igor Pro 6.2.2.2; WaveMetrics Inc., Lake Oswego, USA) was used to fit amplitude histograms to a Gaussian distribution with the distance of the maxima giving the unitary current of one channel opening and cross-checked by direct measurements. The unitary currents were then plotted against the clamped pipette potentials (IV-plot), which were corrected for liquid junction potentials throughout. For symmetrical solutions, the slope of the linear regression equals the conductance. For asymmetrical solutions, GHK theory was used to fit the IV-plot, yielding the permeability for two ions (P_A and P_B) as described in detail in [3].

$$(1) \qquad I = \frac{U \cdot F^2}{R \cdot T} \cdot \left(\frac{P_A \cdot [A]_i + P_B \cdot [B]_i - \ (P_A \cdot [A]_o + (P_B \cdot [B]_o) \cdot \exp\left(-U \cdot \frac{F}{R \cdot T}\right)}{1 - \exp\left(-U \cdot \frac{F}{R \cdot T}\right)} \right)$$

The values for P_x resulting from the fit of currents in asymmetrical solutions were used to predict the conductances (G_x) in symmetrical oocyte Ringer solution at 96 mmol·L^{-1} according to:

$$(2) \qquad G_X = \frac{I}{U} = \frac{F^2}{R \cdot T} \cdot P_X \cdot 96\ \mathrm{mmol{\cdot}L^{-1}}$$

To compare these conductance values with data obtained in a previous study with HEK-293 cells in standard Ringer solutions (145 mmol·L^{-1}) [3], G_x in oocyte solution was multiplied by the concentration ratio yielding:

$$(3)\ G_X\ (145\ \mathrm{mmol{\cdot}L^{-1}}) = G_X\ (96\ \mathrm{mmol{\cdot}L^{-1}}) \times \frac{145\ \mathrm{mmol{\cdot}L^{-1}}}{96\ \mathrm{mmol{\cdot}L^{-1}}}$$

Supplemental Figure

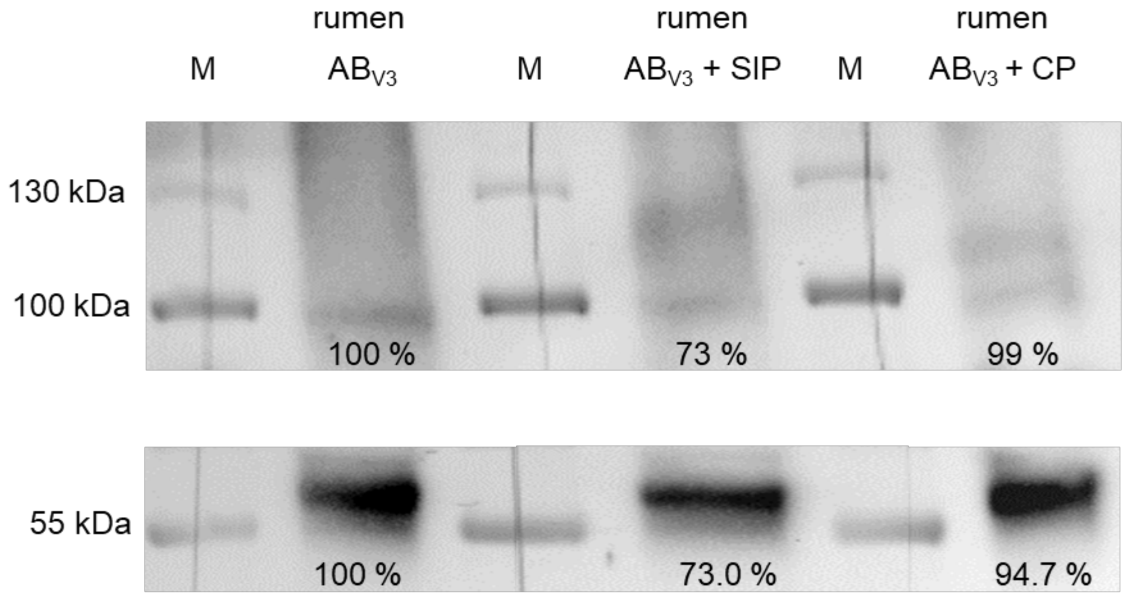

Immunoblot of protein from one animal with a weak band at 90 kDa. Each lane contains 50 µg of ruminal protein (rumen) and is preceded by a marker lane (M). After blotting, the membrane was cut for separate treatment with the TRPV3 antibody (AB$_{V3}$), antibody and specific immunizing peptide (AB$_{V3}$ + SIP), or antibody and control peptide (AB$_{V3}$ + CP). To optimize the exposure time for both of the two bands, all three membrane slices were cut at 72 kDa. The percentages under the lanes represent the relative quantities determinded using ImageLab software (BioRad).

References

1. Abdoun K, Stumpff F, Rabbani I, Martens H (2010) Modulation of urea transport across sheep rumen epithelium in vitro by SCFA and CO2. American journal of physiology Gastrointestinal and liver physiology 298:G190-202. doi:10.1152/ajpgi.00216.2009

2. Barry PH, Lynch JW (1991) Liquid junction potentials and small cell effects in patch-clamp analysis. J Membr Biol 121:101-117. doi:10.1007/BF01870526

3. Schrapers KT, Sponder G, Liebe F, Liebe H, Stumpff F (2018) The bovine TRPV3 as a pathway for the uptake of Na+, Ca2+, and NH4+. PLoS One 13:e0193519. doi:10.1371/journal.pone.0193519
